# Intra-aortic balloon pump does not influence cerebral hemodynamics and neurological outcomes in high-risk cardiac patients undergoing cardiac surgery: an analysis of the IABCS trial

**DOI:** 10.1186/s13613-019-0602-z

**Published:** 2019-11-27

**Authors:** Juliana R. Caldas, Ronney B. Panerai, Edson Bor-Seng-Shu, Graziela S. R. Ferreira, Ligia Camara, Rogério H. Passos, Angela M. Salinet, Daniel S. Azevedo, Marcelo de-Lima-Oliveira, Filomena R. B. G. Galas, Julia T. Fukushima, Ricardo Nogueira, Fabio S. Taccone, Giovanni Landoni, Juliano P. Almeida, Thompson G. Robinson, Ludhmila A. Hajjar

**Affiliations:** 1 Department of Anesthesia, University of São Paulo, São Paulo, São Paulo Brazil; 2 Universidade de Salvador, UNIFACS, Salvador, Bahia Brazil; 3 Department of Cardiovascular Sciences, University of Leicester, Leicester, UK; 4 NIHR Leicester Biomedical Research Centre, Leicester, UK; 5 Department of Neurosurgery, Hospital das Clinicas, University of São Paulo, São Paulo, Brazil; 6 Department of Cardiopneumology, Universidade de São Paulo, São Paulo, Brazil; 7 Critical Care Unit Hospital São Rafael Salvador, Salvador, Brazil; 8 Department of Intensive Care, Hopital Erasme, Brussels, Belgium; 9 Department of Anaesthesia and Intensive Care, IRCCS San Raffaele Scientific Institute and Vita-Salute San Raffaele University of Milan, Milan, Italy; 10 Surgical Intensive Care, Heart Institute, University of São Paulo, Av. Dr. Ene´as de Carvalho Aguiar 44, 05403-000 São Paulo, Brazil; 11 Escola Bahiana de Medicina e Saude Púbica, Salvador, Brazil

**Keywords:** IABP, Transcranial Doppler, Cerebral autoregulation, Cardiopulmonary bypass

## Abstract

**Background:**

The intra-aortic balloon pump (IABP) is often used in high-risk patients undergoing cardiac surgery to improve coronary perfusion and decrease afterload. The effects of the IABP on cerebral hemodynamics are unknown. We therefore assessed the effect of the IABP on cerebral hemodynamics and on neurological complications in patients undergoing cardiac surgery who were randomized to receive or not receive preoperative IABP in the ‘Intra-aortic Balloon Counterpulsation in Patients Undergoing Cardiac Surgery’ (IABCS) trial.

**Methods:**

This is a prospectively planned analysis of the previously published IABCS trial. Patients undergoing elective coronary artery bypass surgery with ventricular ejection fraction ≤ 40% or EuroSCORE ≥ 6 received preoperative IABP (*n* = 90) or no IABP (*n* = 91). Cerebral blood flow velocity (CBFV) of the middle cerebral artery through transcranial *Doppler* and blood pressure through Finometer or intra-arterial line were recorded preoperatively (*T*1) and 24 h (*T*2) and 7 days after surgery (*T*3) in patients with preoperative IABP (*n* = 34) and without IABP (*n* = 33). Cerebral autoregulation was assessed by the autoregulation index that was estimated from the CBFV response to a step change in blood pressure derived by transfer function analysis. Delirium, stroke and cognitive decline 6 months after surgery were recorded.

**Results:**

There were no differences between the IABP and control patients in the autoregulation index (*T*1: 5.5 ± 1.9 vs. 5.7 ± 1.7; *T*2: 4.0 ± 1.9 vs. 4.1 ± 1.6; *T*3: 5.7 ± 2.0 vs. 5.7 ± 1.6, *p* = 0.97) or CBFV (*T*1: 57.3 ± 19.4 vs. 59.3 ± 11.8; *T*2: 74.0 ± 21.6 vs. 74.7 ± 17.5; *T*3: 71.1 ± 21.3 vs. 68.1 ± 15.1 cm/s; *p* = 0.952) at all time points. Groups were not different regarding postoperative rates of delirium (26.5% vs. 24.2%, *p* = 0.83), stroke (3.0% vs. 2.9%, *p* = 1.00) or cognitive decline through analysis of the Mini-Mental State Examination (16.7% vs. 40.7%; *p* = 0.07) and Montreal Cognitive Assessment (79.16% vs. 81.5%; *p* = 1.00).

**Conclusions:**

The preoperative use of the IABP in high-risk patients undergoing cardiac surgery did not affect cerebral hemodynamics and was not associated with a higher incidence of neurological complications.

*Trial registration* http://www.clinicaltrials.gov (NCT02143544).

## Background

Neurological complications remain one of the major hazardous complications of cardiac surgery, despite the continuous evolution of surgical and anesthetic techniques [1, 2]. Although postoperative stroke occurs in up to 6% of the cases, neuropsychological dysfunction occurs much more frequently (40–50% of cases). Additionally, delirium affects up to 70% of high-risk (i.e., combined cardiac surgery procedures with comorbidities and poor ventricular function) patients after cardiac surgery [1–4].

The intra-aortic balloon pump (IABP) is often used to manage patients with advanced heart failure and to preoperatively support high-risk patients by improving myocardial perfusion and lowering cardiac afterload [5–7]. The hemodynamic consequences of the IABP include changes in aortic deflation, an increase in cardiac output, a decrease in systolic and mean arterial pressure and a reduction in systemic vascular resistance [6]. Nevertheless, evidence of the consequences of the IABP on cerebrovascular function is lacking and current knowledge is based mainly on observational studies [7–9].

Studies investigating the effects of the IABP on cerebral circulation provide conflicting results and have suggested that the IABP either decreased, increased or even transiently reversed cerebral blood flow [7–9]. What is clear though is that the use of the IABP can change the temporal pattern of cerebral blood flow (CBF) waveforms during the cardiac cycle, leading to characteristic double-peaked waveforms and the occurrence of transient reversed diastolic (i.e., negative) values of CBF or cerebral blood flow velocity (CBFV) recorded with transcranial Doppler [10]. In addition to these changes in CBF, there are no robust studies on this topic, thus justifying the need for this study.

Previous studies reported improved clinical outcomes with the IABP in patients with aneurysmal subarachnoid and cerebral vasospasm [11, 12], although neurological complications after the IABP, such as delirium, have been described [13, 14]. Moreover, other complications were postulated or confirmed on postmortem examinations and included cerebral air embolism secondary to IABP rupture, medullary ischemia due to mechanical trauma or microemboli and dissecting hematoma of the thoracic aorta [15–18].

We therefore performed a prospective study to assess the effect of the IABP on cerebral hemodynamics and cerebral autoregulation. Furthermore, we evaluated the incidence of neurological complications in high-risk patients after coronary artery bypass graft (CABG) surgery, randomized to receive or not receive preoperative IABP.

## Methods

### Research participants

The present study is a predefined substudy of the single-center, parallel, randomized, clinical trial named Intra-aortic Balloon Counterpulsation in Patients Undergoing Cardiac Surgery (IABCS; ClinicalTrials.gov Identifier: NCT02143544). It was registered in PLATAFORMA LATTES (number 22491913.7.0000.0068), a Brazilian database of clinical trials. The study was performed at the Heart Institute, University of São Paulo. The main IABCS trial was previously published [19].

In the period from May 2014 to June 2016, patients who underwent elective CABG or a combined procedure including CABG with cardiopulmonary bypass (CPB) were eligible to participate in the study if they fulfilled the following criteria: (i) age > 18 years, (ii) European System for Cardiac Operative Risk Evaluation (EuroSCORE) greater than 6 or left ventricular ejection fraction (LVEF) less than 40% on transthoracic echocardiography and (iii) written informed consent. Exclusion criteria were cardiogenic shock, acute myocardial infarction (AMI) less than 48 h prior to enrollment, previous IABP use, AMI mechanical complications, peripheral vascular disease, severe aortic regurgitation, tachyarrhythmia, aortic procedures and coagulopathy (IABCS main study exclusion criteria), pregnancy and absence of temporal bone acoustic windows.

The main study and this substudy were approved by the local ethics committee for the Analysis of Research Projects (CAPPesq) (approval numbers: 835/731 and 0352/08, respectively).

### Randomization and masking

Simple randomization was performed after patient enrollment with a computer-generated list in a 1:1 ratio that was generated online by a Web-based program that ensured allocation concealment. The nature of the intervention precluded blinding of the patients and attending physicians. Outcome assessors were unaware of the assigned treatment.

### Procedure

The IABP was inserted percutaneously through the femoral artery (Sensation 7F; Maquet Cardiopulmonary AG, Mahwah, NJ) after anesthesia induction and immediately before skin incision. Positioning of the IABP was guided by radioscopy. The balloon size was based on the patient’s height. Transesophageal echocardiography was used to confirm correct IABP placement before and after CPB.

Preoperative medication consisted of midazolam (0.1–0.2 mg/kg given orally 30 min before surgery). Anesthesia was induced with fentanyl (3–5 μg/kg), midazolam (0.05 mg/kg), etomidate (0.2–0.3 mg/kg) and pancuronium bromide (0.1 mg/kg). Anesthesia was maintained with isoflurane in oxygen and fentanyl as needed. During CPB, additional doses of midazolam and pancuronium were administered as required.

Systemic and cerebral physiological monitoring was performed at rest in a supine position prior to surgery (*T*1) and at 24 h (*T*2) and 7 days (T3) after surgery. Transcranial *Doppler* (TCD) evaluation of the middle cerebral arteries (MCAs) was carried out using bilateral 2 MHz pulsed range-gated probes (DWL, Doppler-Box, Germany) held in place using a head frame. Insonation depth varied from 50 to 55 mm. If only one MCA could be insonated, this was the side used in subsequent analyses. Time-averaged mean, systolic and diastolic values of blood flow velocities (CBFVm, CBFVs and CBFVd, respectively) and the pulsatility index (PI = CBFVs − CBFVd/CBFVm) were then calculated [20].

Blood pressure was continuously measured noninvasively at *T*1 and *T*3 using finger arterial volume clamping (Finometer PRO; Finapres Medical Systems, Amsterdam, The Netherlands) with the servo-adjust switched off after a stable waveform was achieved with the servo-adjust on. At *T*2, BP was measured invasively (Philips Monitor MP50, Germany) in the radial artery. End-tidal CO_2_ (EtCO_2_) was continuously measured with an infrared capnograph (Dixtal, dx 1265 ETCO_2_ Capnogard, Manaus, Brazil) via a closely fitting mask and was documented at 1-min intervals.

Signals were sampled (100 Hz) and stored for off-line analysis. All recordings were visually inspected, and the BP signal was calibrated using the systolic and diastolic values of radial sphygmomanometry. All signals were filtered with an eighth-order Butterworth low-pass filter with a cut-off frequency of 20 Hz. The beginning and end of each cardiac cycle were detected in the BP signal, and mean BP (MAP), CBFVm, CBFVs, CBFVd and heart rate were obtained for each heartbeat. Beat-to-beat parameters were interpolated with a third-order polynomial and resampled at 5 Hz to generate signals with a uniform time base.

Dynamic cerebral autoregulation (CA) was assessed using transfer function analysis, using spontaneous fluctuations of MAP as input and corresponding changes in CBFVm as output in recordings lasting 5 min, as described previously [21, 22]. The Welch method was adopted for smoothing spectral estimates obtained with the fast Fourier transform (102.4 s segments, 50% superposition) leading to frequency-dependent estimates of coherence, gain and phase. Using the inverse fast Fourier transform, the CBFV response to a step change in MAP was also derived and compared with ten template curves proposed by Tiecks et al. [23] to estimate the autoregulation index (ARI) [21, 24]. Baseline cerebral hemodynamic parameters are reported as the average over a 5-min recording at rest. Impaired dynamic CA was defined as an ARI < 4 [25].

### Assessment of postoperative delirium

From postoperative day 1 until postoperative day 5 or discharge from the ICU, all patients underwent twice-a-day assessments for delirium by the confusion assessment method (CAM), a diagnostic algorithm based on four features: acute change with a fluctuating course, inattention, disorganized thinking and altered level of consciousness [26]. In intubated patients, assessment of delirium was performed with the use of the CAM for the intensive care unit, a validated nonverbal version of the CAM [27].

### Neuropsychological assessment

Two cognitive domains were assessed before surgery and 6 months after surgery. The Mini-Mental State Examination (MMSE) is a simple and standardized test elaborated by Folstein and colleagues to evaluate the cognitive performance of elderly subjects and to quantify cognitive deficits [28]. The MMSE scores range from 0 to 30; abnormal cognition is defined as a score of less than 24. The Montreal Cognitive Assessment (MoCA, version 7.1) was developed to detect mild cognitive impairment (MCI) [29]. The maximal score is 30; scores below 26 indicate abnormal cognition [29]. MCI was defined as MMSE ≤ 24 or MoCA < 26. These tests were administered by a trained nurse who was blinded to all other data and measurements taken in these patients, including allocation to randomized groups.

### Stroke evaluation

The presence of neurological deficit was investigated. Noncontrast computed tomography (CT) scan was the initial imaging modality in the suspicion of acute stroke. Magnetic resonance imaging was performed to confirm the CT scan findings and provide clear evidence of the extent of parenchymal ischemia.

### Statistical analysis

Following assessment of normality with the Kolmogorov–Smirnov (KS) one-sample test, parametric (Student’s *t*) or nonparametric (Mann–Whitney *U*) tests were used as appropriate. Fisher’s exact test was used with categorical variables. The results are expressed as the mean ± standard deviation (SD) or median with interquartile ranges (IQRs). Interhemispherical differences in parameters were tested with the paired Student’s *t* test or Wilcoxon’s nonparametric test. In the absence of differences, values for the right and left MCAs were averaged. Changes in ARI and other parameters at *T*1, *T*2 and *T*3 were assessed with two-way repeated measure ANOVA. In the event of significant effects (either IABP or study time), intergroup differences were assessed with Tukey’s post hoc test. Statistical analyses were performed using SPSS version 22.0 (SPSS Inc., Chicago, IL). A *p*-value < 0.05 was considered statistically significant.

## Results

### Study population

**Fig. 1:**
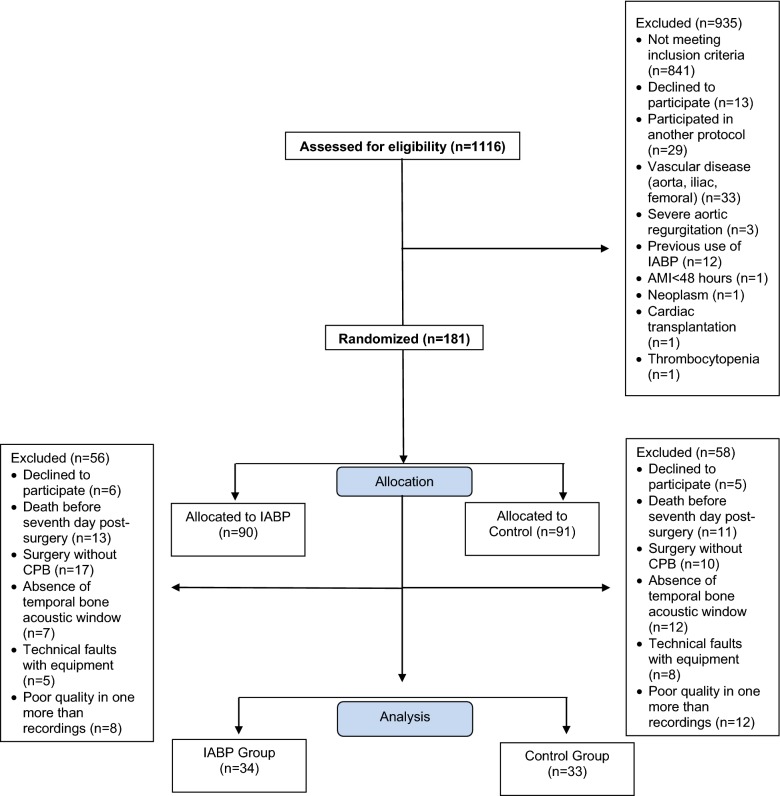
Study flow chart. *IABP* intra-aortic balloon pump, *AI* aortic insufficiency, *AMI* acute myocardial infarction

From the 181 patients included in the IABCS trial between May 2014 and June 2016, we excluded patients who did not survive until the seventh day postsurgery (*n* = 24), who underwent surgery without CPB (*n* = 27), who had absence of temporal bone acoustic windows (*n* = 20), or in the occurrence of technical faults with equipment (*n* = 12), or poor quality in one more recordings (*n* = 21) or patients who declined to participate (10). Of the 67 patients with complete recordings, 34 were included in the IABP group and 33 in the control group (Fig. 1).

Demographics and baseline, surgical and intraoperative characteristics were similar between groups, with the exception of dyslipidemia (higher incidence in control group) and previous myocardial infarction (more prevalent in the IABP group) (Tables 1 and 2). Blood sample tests and systemic hemodynamic parameters are given in Additional file 1: Table S1 for different time periods.

**Table 1:** Demographic and baseline characteristics

Variable	IABP *n* = 34	CG *n* = 33	*p*-value
Gender, male	27 (79.4%)	24 (72.7%)	0.520
Age, years	64 ± 8	64 ± 10	0.750
LVEF (%)	40 [36–42]	40 [33–45]	0.455
EuroSCORE	6 [4–7]	5 [3–6]	0.158
Risk factors			
Previous cardiac surgery, *n*	0	0	–
Previous myocardial infarction, *n* (%)	31 (91.2%)	24 (72.7%)	0.049
Hypertension, *n* (%)	26 (76.5%)	27 (81.8%)	0.427
Peripheral vascular disease, *n* (%)	5 (14.7%)	2 (6.1%)	0.197
COPD, *n* (%)	1 (2.9%)	1 (3%)	1.000
Current smoking, *n* (%)	8 (23.5%)	8 (24.2%)	0.945
Previous smoking < 6 months, *n* (%)	14 (41.2%)	18 (54.5%)	0.273
Dyslipidemia, *n* (%)	19 (55.9%)	27 (81.8%)	0.022
Diabetes, *n* (%)	16 (47.1%)	17 (51.5%)	0.715
Atrial fibrillation, *n* (%)	3 (8.8%)	2 (6.1%)	1.000
Previous stroke, *n* (%)	4 (11.8%)	1 (3.0%)	0.356
Hepatic disease, *n* (%)	0	0	–
Obesity (BMI > 30) *n* (%)	3 (8.8%)	7 (21.2%)	0.186
Left coronary trunk lesion > 50%, *n* (%)	10 (29.4%)	11 (33.3%)	0.729
Valve disease, *n* (%)	6 (17.6%)	3 (9.1%)	0.476
Medication			
Beta-blocker, *n* (%)	25 (73.5%)	29 (87.9%)	0.138
ACE inhibitor, *n* (%)	23 (67.6%)	27 (81.8%)	0.183
Acetylsalicylic acid, *n* (%)	26 (76.5%)	28 (84.8%)	0.539
Vitamin K antagonist, *n* (%)	1 (2.94%)	2 (6.1%)	0.614

Values are n (%), population mean ± SD or median [interquartile range]. *IABP* intra-aortic balloon pump, *CG* control group, *LVEF* left ventricular ejection fraction, *BMI* body mass index, *ACE* angiotensin-converting enzyme, *CABG* coronary artery bypass graft, *CPB* cardiopulmonary bypass, *COPD* chronic obstructive pulmonary disease

**Table 2:** Intraoperative data

Variable	IABP (*n* = 34)	Control (*n* = 33)	*p* value
Surgery type			
Isolated CABG	32	32	1.000
No. of grafts			0.110
1	0	1	
2	9	5	
3	10	19	
4	13	7	
5	2	1	
Duration of surgery, h	5.1 [4.4–5.6]	5.0 [4.3–6.0]	0.594
Duration of CPB, min	95 [75–113]	92 [83–117]	0.697
Aortic cross-clamp time, min	75 [59–90]	74 [58–90]	0.874
RBC transfusion	16 (47.1)	8 (24.2)	0.051
Antifibrinolytic	32 (94.1)	28 (87.5)	0.420
Crystalloid, mL	2000 ([500–2500]	2500 [500–3250]	0.094

*IABP* intra-aortic balloon pump, *CABG* coronary artery bypass grafting, *LIMA* left internal mammary artery, *CPB* cardiopulmonary bypass

Data are presented as *n* (%) of patients or median (interquartile range)

### Physiological and laboratory values

Hemodynamic parameters, hemoglobin, blood lactate, base excess, mixed venous saturation and venous–arterial CO_2_ tension gap were not different between the groups (Additional file 1: Table S1).

### Peripheral hemodynamic parameters

**Fig. 2:**
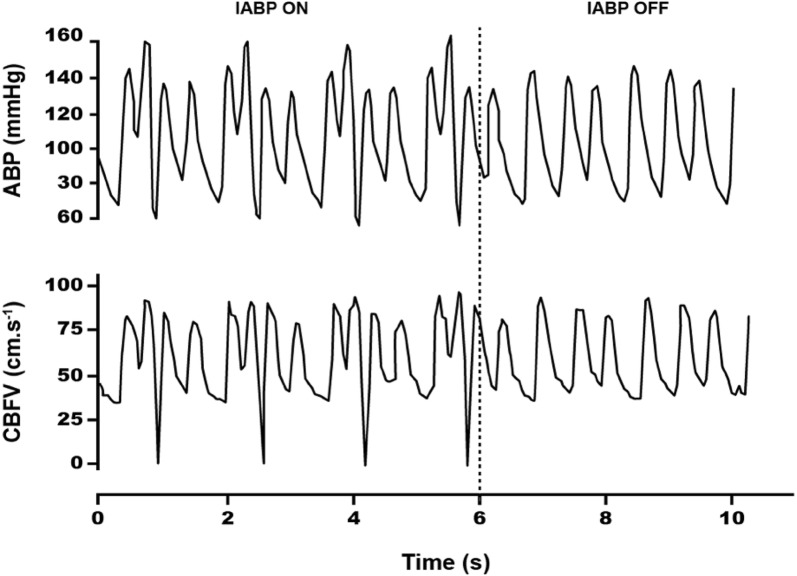
Ten-second continuous recording of blood pressure and cerebral blood flow velocity from 63-year-old male patient with IABP ratio 1:3, showing removal of the intra-aortic balloon pump at *t* = 6 s. This subject is representative of the frequent occurrence of cerebral blood flow velocity diastolic values that are negative or near zero following deflation of the balloon (Reproduced with permission from Caldas et al. [22])

A representative recording of BP and CBFVm, indicating the moment of balloon withdrawal, is given in Fig. 2, showing the altered cardiac cycle patterns resulting from inflation and deflation of the IABP with a 1:3 ratio.

All peripheral and cerebral hemodynamic parameters were different between baseline and 7 days after surgery (Table 3), but the only difference between the groups was a lower diastolic BP in the IABP group compared to the control group at *T*2 (*p* = 0.003)

**Table 3:** Systemic and cerebral hemodynamic parameters

Variable	IABP group (*N* = 34)			Control group (*N* = 33)			*p*-value effect IABP	*p*-value effect
	*T*1	*T*2	*T*3	*T*1	*T*2	*T*3		*T*1–*T*3
EtCO_2_ (mmHg)	33.1 ± 3,6	33.1 ± 3.2	31.1 ± 3.4^*^	34.7 ± 4.9	34.2 ± 4.0	33.0 ± 3.1^*^	0.809	0.004
Mean BP (mmHg)	91.1 ± 13.3	83.2 ± 13.4^*^	89.0 ± 9.1	93.5 ± 13.6	80.4 ± 12.1^*^	90.3 ± 8.3	0.853	< 0.001
Systolic (BP mmHg)	132.9 ± 20.3	127.0 ± 23.6^*^	127.3 ± 18.3^*^	138.1 ± 22.7	128.2 ± 17.2^*^	125.8 ± 16.8^*^	0.629	0.007
Diastolic BP (mmHg)	67.9 ± 10.9	46.2 ± 13.9^*#^	67.9 ± 8.7^*^	69.4 ± 10.7	58.6 ± 11.7^*^	70.7 ± 8.6^*^	0.003	< 0.001
HR (bpm)	68.4 ± 11.4	103.8 ± 17.0^*^	89.2 ± 15.9^*^	61.9 ± 9.1	101.9 ± 13.0^*^	90.5 ± 12.5^*^	0.304	< 0.001
ARI	5.5 ± 1,9	4.0 ± 1.9^*^	5.7 ± 2.0	5.7 ± 1.7	4.1 ± 1.6^*^	5.7 ± 1.6	0.978	< 0.001
ARI < 4 (*n*, %)	7 (20.6%)	18 (52.9%)^*^	7 (20.5%)	5 (15.1%)	17 (51.5%)^*^	4 (12.1%)	0.138	< 0.001
CBFV MCA (cm/s)	57.3 ± 19.4	74.0 ± 21.6^*^	71.1 ± 21.3^*^	59.3 ± 11.8	74.7 ± 17.5^*^	68.1 ± 15.1^*^	0.952	< 0.001
Systolic CBFV (cm/s)	89.1 ± 28.5	107.6 ± 32.5^*^	109.6 ± 30.2^*^	90.6 ± 20.5	113.3 ± 24.7^*^	101.1 ± 22.2^*^	0.942	< 0.001
Diastolic CBFV (cm/s)	39.9 ± 11.4	33.2 ± 14.4^*#^	49.0 ± 21.4	39.7 ± 8.2	51.5 ± 13.8	48.8 1 ± 0.8	0.001	< 0.001
PI	0.82 ± 0.15	1.03 ± 0.26^*#^	0.85 ± 0.16	0.85 ± 0.17	0.84 ± 0.17	0.80 ± 0.18	0.001	< 0.001

Values are population mean ± SD. *IABP* intra-aortic balloon pump, *EtCO*_*2*_ end-tidal CO_2_, *BP* blood pressure, *HR* heart rate, *CBFV* cerebral blood flow velocity, *MCA* middle cerebral artery, *PI* pulsatility index, *T1* assessment before surgery, *T2* assessment 24 h after surgery, *T3* assessment 7 days after surgery

^#^*p* < 0.05 vs. controls; *p < 0.05 vs. time (repeated measures ANOVA)

### Cerebral autoregulation and cerebral hemodynamic parameters

No significant differences between the right and left MCAs were observed for the cerebral hemodynamic parameters, and therefore, values were averaged for further analyses.

**Fig. 3:**
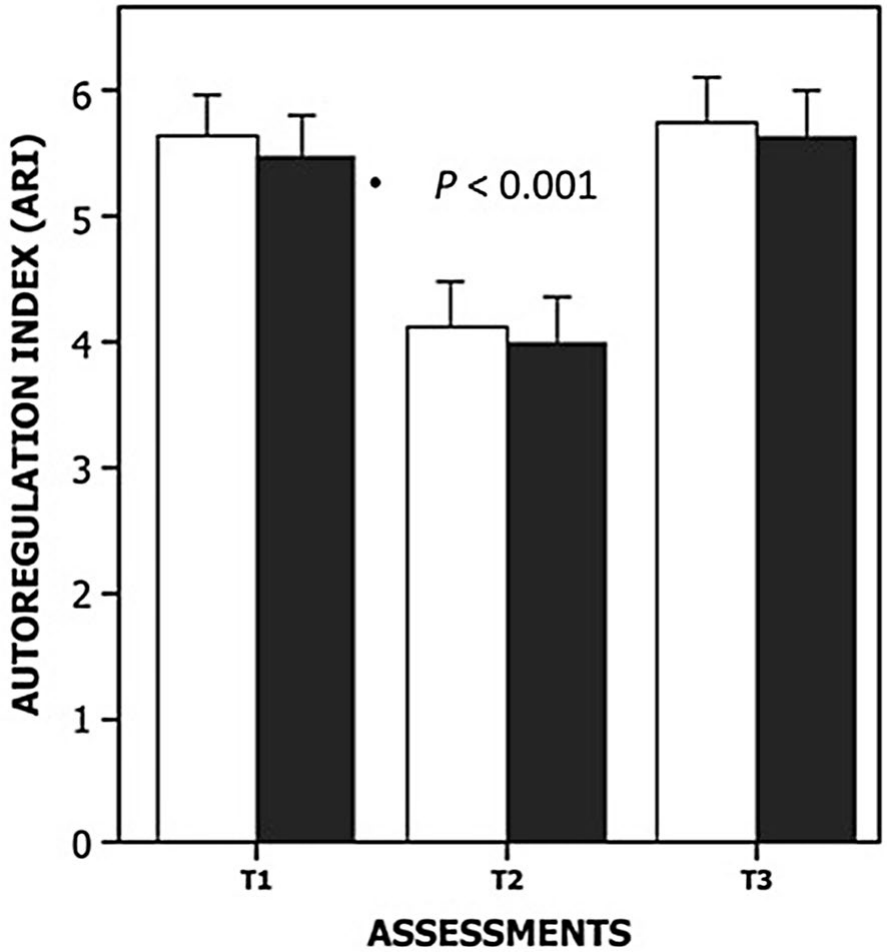
Mean values (± 1 standard error) of ARI for control group population (white bar) and intra-aortic balloon pump population (black bar), followed by the session number corresponding to before the surgery (*T*1), 24 h (*T*2) and 7 days (*T*3) of follow-up

**Fig. 4:**
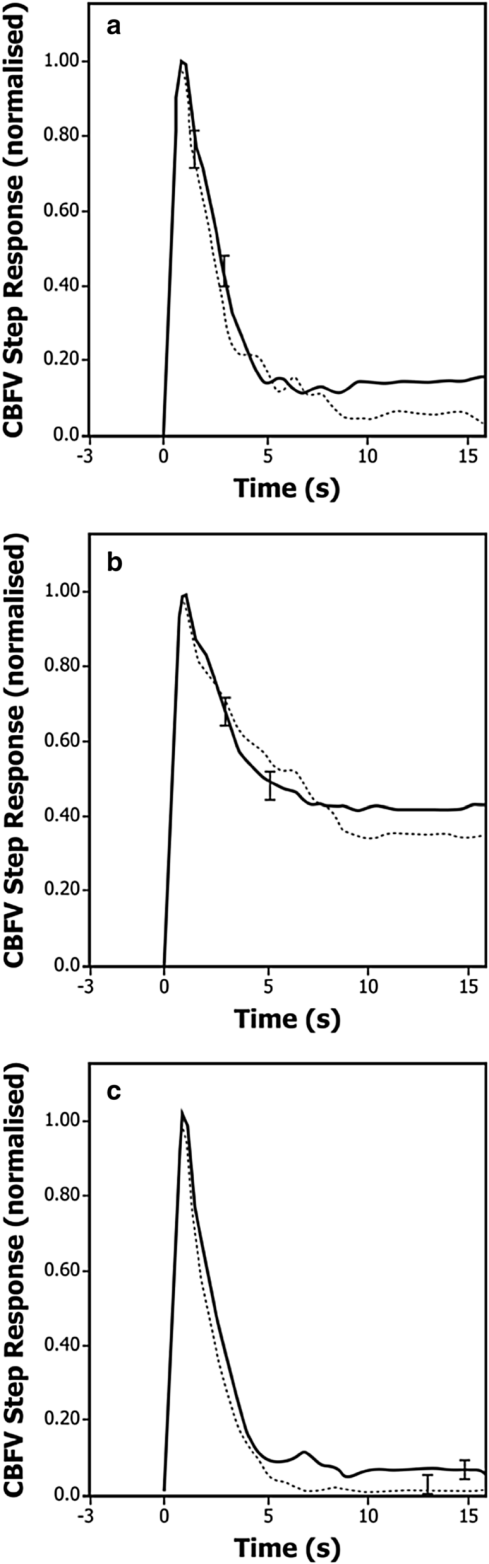
Control group (dotted line) and intra-aortic balloon pump group (continuous line) population averages of cerebral blood flow velocity step response. Before surgery (**a**) and 24 h (**b**) and 7 days after surgery (**c**). Largest ± 1 standard error is represented at the point of occurrence

CBFV, ARI and PI were influenced by the timing of measurement (Figs. 3 and 4, Table 3); in particular, CBFV was depressed at *T*1 as compared to *T*2 and *T*3 (*p* < 0.0001), and ARI was depressed at *T*2 in comparison with *T*1 and *T*3 (*p* < 0.0001), irrespective of the randomized group (Table 3, Figs. 3 and 4). Patients from both groups presented with more impairment of dynamic CA at *T*2 when compared to *T*1 and *T*3. The only difference between groups was observed for PI, which was higher in the IABP group at *T*2 in comparison with the control group (*p* = 0.001) (Table 3). Six patients in the IABP group showed reversal of the CBFV waveform.

### Neurological complications

There was no difference in the rate of postoperative delirium, seizure or stroke between the groups. Six months after surgery, 24 (71%) patients returned for neurocognitive assessment in the IABP group and 27 (82%) returned in the control group, without any differences being observed by randomized group for either MMSE or MoCA (Table 4).

**Table 4:** Occurrence of neurological complications

Variable	IABP group (*N* = 34)	Control group (*N* = 33)	*p*-value
Delirium (*n*, %)	9 (26.5%)	8 (24.2%)	0.834
Seizure	1 (2.9%)	1 (3%)	1.000
Stroke	1 (3.0)	1 (2.9%)	1.000
Presurgery			
MoCA	21 [18–24]	21 [5–25]	0.667
MoCA < 26 (*n*, %)	30 (88.2%)	28 (84.8%)	1.000
MMSE	25 [23–28.5]	25 [23–28.5]	0.632
MMSE ≤ 24 (*n*, %)	13 (39.4%)	14 (42.4%)	1.000
Six months postsurgery (*N* = 24)		(*N* = 27)	
MoCA	24.0 [20.2–25.0]	20.0 [17–24.0]	0.079
MoCA < 26 (*n*, %)	19 (79.1%)	22 (81.5%)	1.000
MMSE	28.0 [25.2–29.0]	26 [23.0–29.0]	0.216
MMSE ≤ 24 (*n*, %)	4 (16.7%)	11 (40.7%)	0.073

Data are *n* (%) or median [interquartile range]

*IABP* intra-aortic balloon pump, *CG* control group, *MMSE* Mini-Mental State Examination, *MoCA* Montreal Cognitive Assessment

## Discussion

### Main findings

This is the first study to analyze the effects of the IABP on cerebral hemodynamics through serial evaluations of dynamic CA with the use of transcranial Doppler in high-risk patients undergoing cardiac surgery with CPB. Neurological complications are frequent complications after cardiac surgery, resulting in higher mortality rates and worse long-term outcomes. Our data demonstrate that the use of the IABP does not affect cerebral hemodynamics in high-risk patients undergoing CABG with CPB. Furthermore, the use of the IABP did not increase the incidence of postoperative delirium, stroke or cognitive decline 6 months after surgery. These results suggest the use of the IABP does not contribute to the occurrence of neurological complications after cardiac surgery.

### IABP and cerebral hemodynamics

Our study adds to the existing literature about the effects of the IABP on cerebral hemodynamics. Since its introduction in 1960, the IABP has become of the most used assist devices in clinical practice [30]. The benefit of the IABP on systemic hemodynamics results from the reduced afterload in systole and increased coronary perfusion during diastole, leading to improvements in cardiac output, especially in ischemic cardiac patients [31]. However, previous studies that assessed cerebral hemodynamics in patients with the use of the IABP have reported conflicting results. Pfluecke et al. [9] demonstrated that the use of the IABP improved CBF, particularly in patients with preexisting heart failure. In 2014, Yang et al. [32] evaluated 12 patients who required the IABP and extracorporeal membrane oxygenation (ECMO) after cardiac surgery and reported that CBF changes were dependent on IABP, with CBFV decreasing during myocardial stunning but increasing during recovery of cardiac function. Other studies in patients following cardiac surgery showed that the use of the IABP modified the temporal pattern of the CBF waveform but without changing the mean value of CBFV [7, 8]. In cardiogenic shock, the IABP was shown not to affect the microcirculation in patients undergoing ECMO when brain oxygenation was assessed with near-infrared spectroscopy [33]. Overall, our data showed no difference in mean bilateral CBFV of the MCAs between the two groups, despite the altered CBFV waveforms observed in the IABP group. Uniquely, our study randomized patients to the IABP and additionally included an assessment of dynamic CA.

A small (*n* = 20) observational study of dynamic CA with IABP assessed CA by the time delay between fluctuations in CBFV and BP, estimated using a cross-correlation analysis technique [34]. Unfortunately, the use of cross-correlation for assessment of dynamic CA is not as well validated as TFA [35], with very limited information available to allow interpretation of time-delay data, including ranges of normality. Nevertheless, the results reported by Bellapart et al. [34] are in agreement with ours.

The IABP led to changes in diastolic BP, explaining the changes in CBFV waveforms that were also reported in other studies [7, 8]. The alterations in CBFV diastolic values in our study resulted in increases in PI in the IABP group. A small subgroup of these patients (17%) showed reversal of the CBFV waveform during the cardiac cycle. This phenomenon changes the PI value because of the negative values of diastolic velocity, and for this reason, PI becomes meaningless in this small number of patients. Further work is needed in a larger population of patients with CBFV waveform reversal to allow more in depth analysis of the consequences of this phenomenon. Changes in PI were first reported in 1986, shortly after TCD was introduced [36]. In 2004, Bellner et al. concluded that PI can serve as a surrogate for estimating elevated ICP [37] or an increase in cerebrovascular resistance [37, 38] on normocapnia and normal BP. In our patients due the changes on diastolic BP, PI may have lost the ability to be interpreted as a surrogate of elevated ICP. On the other hand, an elevated PI has long been acknowledged to be a nonspecific finding, and the PI may be heavily influenced by the MAP and PaCO_2_ [38, 39]. Moreover, in a study of TBI [40], the reliability of PI, as a predictor of ICP, did not improve even when hemodynamic variables were adjusted, thus suggesting that the PI may be of limited use in clinical practice.

### Effect of IABP on clinical outcomes

Regarding clinical outcomes, our study is a substudy of a recent clinical trial reporting that preoperative use of the IABP in high-risk patients did not change clinical outcomes [19].

Previous studies assessing the effects of cardiac surgery on CBFV and CA [41, 42] showed that CA may be impaired during cardiac surgery [43]. However, our results showed that CA impairment occurs not only during surgery but also 24 h postsurgery in most patients (57%). Noteworthy, the use of the IABP did not protect the brain from worsening of dynamic CA in the immediate postoperative period.

In patients undergoing cardiac surgery, postoperative brain injury significantly contributes to increased morbidity and mortality and has negative consequences on quality of life and healthcare costs [2]. The most commonly encountered neurological injuries are postoperative delirium, cognitive decline and stroke [4]. Delirium is an acute syndrome affecting awareness and attention with a fluctuating course, which is extremely common after cardiac surgery [2, 44]. It was associated with impaired cognitive function and increased morbidity, length of stay and mortality [44, 45]. The use of the IABP has been described as a risk factor for delirium after cardiac surgery [13, 46]. In our study, the incidence of delirium after surgery was 25% and similar between groups. This incidence is relatively low compared to other reports in the literature mentioning rates up to 70% for high-risk patients after cardiac surgery [4, 45]. The relatively low rate of delirium in our case could be attributed to adherence to strict surgical guidelines and protocols for postoperative nursing care in our service. However, the diagnosis of delirium is based on clinical signs observed by doctors and nurses and has low sensitivity and the underdiagnosis of hypoactive forms of delirium might occur [47]. Stroke continues to be one of the most debilitating and devastating complications of cardiac surgery.

Six months after the surgery, we did not find a significant decrease in the scores of the cognitive scales, and this was not different between the groups. There was a trend toward cognitive decline in the MMSE scale in the control group, but his result was not confirmed by the number of patients who had MoCA scale < 26. The causes of cognitive decline after cardiac surgery remain unclear. A recent study [48] confirmed that cerebral microbleeds detected in MRI (silent stroke) are common in postoperative cardiac surgery, with 76% of authors finding a prevalence of 44% of neuropsychological impairment, although not associated with microbleeds [48]. Despite trends of cognitive decline in the control group, we also did not find a relationship with cerebral hemodynamics in that case. On the other hand, we did not use the tests recommended by the Statement of Consensus on Assessment of Neurobehavioral Outcome after Cardiac Surgery, such as Trail Making Tests (parts A and B) and the Grooved Pegboard Test [49].

The prevalence of postoperative cognitive decline is approximately 25% to 50%, and it was previously demonstrated that there is a temporal course in neurocognitive dysfunction after cardiac surgery [4, 50]. Although some studies reported that many patients with postoperative cognitive decline will improve 3 months after surgery, this results in a tremendous burden for patients, caregivers and society [51, 52].

### Limitations of the study

Our study has limitations. TCD cannot provide absolute measurements of CBF, and the use of CBFV as a surrogate relies on the assumption that the MCA diameter remains approximately constant. This is likely to be the case during the 5-min baseline measurements obtained at rest, with stable values of PaCO_2_ [53]. Nevertheless, differences in insonation angle, the chance of arteries other than the MCA being insonated and intersubject anatomical differences, including the acoustic permeability of temporal windows, are factors that need to be considered as potential limitations. However, the high temporal resolution of TCD is paramount to allow the analysis of transient CBFV responses to spontaneous changes in blood pressure [53]. Differences in the method of measurement of ABP, with the Finometer being used at *T*1 and *T*3 and an arterial line at *T*2, could also be seen as a potential limitation. However, a detailed previous study showed excellent agreement between the two methods for assessment of dynamic CA [54]. Additionally, one of the strengths of our study is that we performed a serial analysis of cerebral circulation, with a total of 201 recordings of 67 patients. In assessing the effects of IABP on BP and CBFV, we expressed diastolic values at their usual point of occurrence that is the end of diastole. In doing so, we followed the literature and also highlighted the most relevant hemodynamic changes induced by the cycling of balloon inflation/deflation [22] resulting in the differences shown in Table 3. On the other hand, this choice does not reflect the potential benefits of IABP to improve coronary artery perfusion that are likely to be better expressed by averaged values over the entire duration of diastole. Although not directly related to the main hypothesis of our study, this is something important to bear in mind when interpreting the results in Table 3. Finally, the MRI was performed only if there was the suspicion of an acute stroke; for this reason, we might have missed silent strokes.

In addition, we excluded from our analysis 11 patients who died within 7 days after surgery. This could have influenced our results. However, in the main study—the IABCS trial—we performed an intention to treat analysis and we did not find any difference in outcomes between groups.

## Conclusions

The present study demonstrated that IABP use in high-risk cardiac patients undergoing cardiac surgery with CPB does not adversely affect cerebral hemodynamics and does not influence the occurrence of neurological complications, including delirium, stroke and cognitive decline.

## Supplementary information

**Additional file 1**: Table S1. Blood test and systemic hemodynamic parameters during surgery and at the first day in the ICU.

## Data Availability

All data generated or analyzed during this study are included in this published article its additional files.

## Abbreviations

ACE: angiotensin-converting enzyme
ARB: angiotensin receptor blocker
ANOVA: analysis of variance
AKI: acute kidney injury
ARDS: acute respiratory distress syndrome
ARI: autoregulation index
BMI: body mass index
BP: blood pressure
CA: cerebral autoregulation
CABG: coronary artery bypass graft
CBF: cerebral blood flow
CBFV: cerebral blood flow velocity
CBFVm: cerebral blood flow velocity mean
CBFVs: cerebral blood flow velocity systolic
CBFVd: cerebral blood flow velocity diastolic
CAM: ICU Confusion Assessment Method for the ICU
CO_2_: carbon dioxide
CPB: cardiopulmonary bypass
CT: computed tomography
ECMO: extracorporeal membrane oxygenation
EtCO_2_: end-tidal CO_2_
EuroSCORE: European System for Cardiac Operative Risk Evaluation
HR: heart rate
IABP: intra-aortic balloon pump
ICU: intensive care unit
IQR: interquartile range
LVEF: left ventricle ejection fraction
MAP: mean blood pressure
MCA: middle cerebral artery
MCI: mild cognitive impairment
MMSE: Mini-Mental State Examination
MoCA: Montreal Cognitive Assessment
PI: pulsatility index
SD: standard deviation
TCD: transcranial Doppler
TFA: transfer function analysis
