# Supplementary material for: Intra-aortic balloon pump does not influence cerebral hemodynamics and neurological outcomes in high-risk cardiac patients undergoing cardiac surgery: an analysis of the IABCS trial
Source: Ann Intensive Care. 2019 Nov 27;9:130. doi: 10.1186/s13613-019-0602-z (PMC6879692; doi:10.1186/s13613-019-0602-z)
Supplement: Supplementary file 1 — Additional file 1: Table S1. Blood test and systemic hemodynamic parameters during surgery and at the first day in the ICU. [file 13613_2019_602_MOESM1_ESM.docx]

Additional file 1

Intra-aortic balloon pump does not influence cerebral hemodynamic and neurological outcomes in high-risk cardiac patients undergoing cardiac surgery: an analysis of the IABCS trial

^1,2,7^Juliana R Caldas, MD, PhD; ^3,4^Ronney B Panerai, PhD; ^5^Edson Bor-Seng-Shu, MD, PhD; ^6^Graziela SR Ferreira, MD, PhD; ^1^Ligia Camara, BSc; ^7^Rogério H. Passos, MD; ^5^Angela M Salinet, PhD; ^5^Daniel S Azevedo, MD; ^5^Marcelo de-Lima-Oliveira, MD; ^1^Filomena RBG Galas MD, PhD; ^5^Ricardo Nogueira, MD, PhD; ^8^Fabio S Taccone, MD, PhD; ^9^Giovanni Landoni, MD, PhD; ^1^Juliano P Almeida, MD, PhD; ^3,4^Thompson G Robinson, MD; ^6^Ludhmila A Hajjar, MD, PhD.

Table S1. Blood test and systemic hemodynamic parameters during surgery and at the first day in the ICU

| Variable | IABP | CONTROL | p |
| --- | --- | --- | --- |
|  | n = 34 | n = 33 |  |
| **BEFORE CPB** |  |  |  |
| Heart rate (bpm) | 67 ± 11 | 68 ± 13 | 0.639 |
| Hemoglobin (g/L) | 12.4 ± 1.9 | 13.2 ± 11.7 | 0.126 |
| pH | 7.41 ± 0.05 | 7.42 ± 0.05 | 0.782 |
| PaO_2_ (mmHg) | 212.45 ± 61.59 | 240.9 ± 67.25 | 0.052 |
| SatO_2_ | 100 (99 - 100) | 99 (98 - 100) | 0.431 |
| PvO_2_ (mmHg) | 42 (38 - 48) | 43 (38 - 46) | 0.620 |
| SvO_2_ (%) | 74.22 ± 9.92 | 74.7 ± 7.5 | 0.567 |
| GAP CO_2_ (mmHg) | 5.8 ± 3.88 | 7.66 ± 4.6 | 0.108 |
| Lactate (mg/dL) | 13 (11 - 17) | 12 (10 - 14) | 0.381 |
| CVP (mmHg) | 11.11 ± 3.71 | 11.09 ± 5.81 | 0.058 |
| Cardiac index (L/min/m^2^) | 2.0 (1.8 - 2.5) | 2.1 (1.8 - 2.6) | 0.584 |
| Cardiac output (L/min) | 3.8 (3.3 - 4.23) | 3.9 (3.15 - 4.98) | 0.804 |
| MAP (mmHg) | 69.91 ± 7.13 | 69.71 ± 9.09 | 0.924 |
| **AFTER CPB** |  |  |  |
| Heart rate (bpm) | 94 ± 14 | 94 ± 15 | 0.632 |
| Hemoglobin (g/L) | 9.28 ± 1.56 | 10.05 ± 1.43 | 0.518 |
| pH | 7.37 ± 0.04 | 7.36 ± 0.04 | 0.260 |
| PaO_2_ (mmHg) | 167.18 ± 50.37 | 138.72 ± 43.52 | 0.285 |
| SatO_2_ | 99 (98 - 100) | 98 (97 - 99) | 0.132 |
| PvO_2_ (mmHg) | 48 (41 - 55) | 47 (44 - 59) | 0.660 |
| SvO_2_ (%) | 78.47 ± 10.04 | 79.42 ± 7.46 | 0.893 |
| GAP CO_2_ (mmHg) | 25 (22 - 30) | 26 (22 - 33) | 0.277 |
| Lactate (mg/dL) | 4.57 ± 2.11 | 5.66 ± 4.32 | 0.676 |
| CVP (mmHg) | 11.86 ± 4.03 | 10.5 ± 4.46 | 0.354 |
| Cardiac index (L/min/m^2^) | 2.4 (2.2 - 3) | 2.6 (2.2 - 2.9) | 0.945 |
| Cardiac output (L/min) | 4.65 (4.05 - 5.65) | 4.7 (3.7 - 7.48) | 1.000 |
| MAP (mmHg) | 69.9 ± 7.1 | 69.7 ± 9.1 | 0.924 |
| **ICU Admission** |  |  |  |
| Heart rate (bpm) | 101 ± 15 | 103 ± 14 | 0.632 |
| Hemoglobin (g/L) | 11.08 ± 1.21 | 11.3 ± 1.4 | 0.518 |
| pH | 7.36 ± 0.05 | 7.36 ± 0.06 | 0.260 |
| PaO_2_ (mmHg) | 164.5 ± 36.5 | 160.0 ± 40.4 | 0.285 |
| SatO_2_ | 99 (98 - 99) | 99 (98 - 99) | 0.768 |
| PvO_2_ (mmHg) | 41 (32 - 47) | 41 (35 - 49) | 0.797 |
| SvO_2_ (%) | 71.12 ± 12.31 | 69.3 ± 11.0 | 0.893 |
| GAP CO_2_ (mmHg) | 21 (15 - 31) | 24 (16 - 35) | 0.127 |
| Lactate (mg/dL) | 5.8 ± 4.0 | 5.7 ± 2.5 | 0.676 |
| CVP (mmHg) | 8.1 ± 3.7 | 11.0 ± 4.6 | 0.354 |
| Cardiac index (L/min/m^2^) | 2.2 (2 - 2.8) | 2.7 (1.9 – 3.0) | 0.338 |
| Cardiac output (L/min) | 3.8 (3.5 - 5.3) | 4.8 (3.1 - 5.5) | 0.350 |
| MAP (mmHg) | 92.9 ± 16.8 | 89.3 ± 12.7 | 0.341 |
| **12 h ICU** |  |  |  |
| Heart rate (bpm) | 99 ± 18 | 103 ± 15 | 0.632 |
| Hemoglobin (g/L) | 10.7 ± 1.19 | 11.3 ± 1.8 | 0.518 |
| pH | 7.37 ± 0.05 | 7.37 ± 0.04 | 0.260 |
| PaO_2_ (mmHg) | 123.0 ± 34.4 | 120.3 ± 27.1 | 0.285 |
| SatO_2_ | 98 (97 - 99) | 98 (97 - 99) | 0.918 |
| PvO_2_ (mmHg) | 37 (34 - 45) | 37 (33 - 40) | 0.229 |
| SvO_2_ (%) | 66.16 ± 8.27 | 65.3 ± 11.0 | 0.893 |
| GAP CO_2_ (mmHg) | 19 (14 - 34) | 26 (15 - 39) | 0.127 |
| Lactate (mg/dL) | 6.48 ± 4.22 | 5.98 ± 2.87 | 0.676 |
| CVP (mmHg) | 8.9 ± 3.6 | 10.1 ± 3.6 | 0.354 |
| Cardiac index (L/min/m^2^) | 2.6 (2.1 - 3.1) | 2.8 (2.3 - 3.2) | 0.446 |
| Cardiac output (L/min) | 4.15 (3.73 - 5.48) | 5.0 (3.5 - 6.5) | 0.573 |
| MAP (mmHg) | 83.0 ± 10.2 | 88.6 ± 13.7 | 0.073 |
| **24 h ICU** |  |  |  |
| Heart rate (bpm) | 106 ± 16 | 103 ± 14 | 0.632 |
| Hemoglobin (g/L) | 10.1 ± 1.5 | 10.7 ± 2.3 | 0.518 |
| pH | 7.4 ± 0.07 | 7.4 ± 0.04 | 0.260 |
| PaO_2_ (mmHg) | 114.7 ± 56.7 | 103.6 ± 34.2 | 0.285 |
| SatO_2_ | 97.3 ± 1.3 | 98.8 ± 1.5 | 0.440 |
| PvO_2_ (mmHg) | 35.8 ± 4.4 | 40.3 ± 8.7 | 0.550 |
| SvO_2_ (%) | 67.4 ± 10.6 | 68.4 ± 10.4 | 0.893 |
| GAP CO_2_ (mmHg) | 33.6 ± 23.3 | 24.4 ±17.5 | 0.431 |
| Lactate (mg/dL) | 8.2 ± 5.2 | 5.1 ± 3.6 | 0.676 |
| CVP (mmHg) | 8.7 ± 3.5 | 9.3 ± 3.2 | 0.354 |
| Cardiac index (L/min/m^2^) | 2.8 (2.5 – 3.2) | 2.8 (2.5 - 3.3) | 0.796 |
| Cardiac output (L/min) | 4.8 (4.2 – 5.9) | 5.4 (4.7 - 6.5) | 0.340 |
| MAP (mmHg) | 85.63 ± 14.03 | 80.16 ± 10.03 | 0.081 |

ICU, intensive care unit; PaO_2_, arterial oxygen tension; SatO_2_, arterial oxygen saturation; BE, base excess; PvO_2_, venous oxygen tension; SvO_2_, saturation from mixed venous blood; GAP CO_2_, central venous-to-arterial CO_2_ difference; CO_2_, carbon dioxide; CVP, central venous pressure; DO_2_, oxygen delivery; VO_2,_ oxygen consumption; MAP, mean arterial pressure.
